# Developmental Competence and Pluripotency Gene Expression of Cattle Cloned Embryos Derived from Donor Cells Treated with 5-aza-2'-deoxycytidine

**Published:** 2011-02-20

**Authors:** Farnoosh Jafarpour, Sayed Morteza Hosseini, Mahdi Hajian, Mohsen Forouzanfar, Parvaneh Abedi, Laleh Hosseini, Somaye Ostadhosseini, Soghra Gholami, Mohammad Hossein Nasr Esfahani

**Affiliations:** 1 Department of Anatomy and Embryology, School of Veterinary Medicine, Shiraz University, Shiraz, Iran; 2 Department of Reproduction and Development, Reproductive Biomedicine Center, Royan Institute for Animal Biotechnology, ACECR, Isfahan, Iran; 3 School of Sciences, Islamic Azad University, Marvdasht Branch, Marvdasht, Iran

**Keywords:** Epigenesis, Nuclear Reprogramming, Nuclear Transfer

## Abstract

**Background:**

Reconstructed embryos from terminally differentiated somatic cells have revealed high levels of genomic methylation which results in inappropriate expression patterns of imprinted and non-imprinted genes. These aberrant expressions are probably responsible for different abnormalities during the development of clones. Improvement in cloning competency may be achieved through modification of epigenetic markers in donor cells.

**Materials and Methods:**

Our objective was to determine if treatment of donor cells for 72 hours with 5-aza-2'-deoxycytidine (5-aza-dc; 0-0.3 μM), a DNA methyl transferase inhibitor, improved development and expression of Oct-4.

**Results:**

In comparison with untreated cells, 0.01 and 0.08 μM 5-aza-dc treated cells insignificantly decreased the blastocyst rate (32.1% vs. 28.6% and 27.2%, respectively) while it was significant for 0.3 μM treated cells (6.5%). Embryo quality as measured by the total cell number (TCN) decreased in a dose-related fashion, which was significant at 0.08 and 0.3 μM 5-aza-dc treated cells when compared with 0 and 0.01 μM 5-aza-dc treated cells. Although reconstructed embryos from 0.08 and 0.3 μM 5-aza-dc treated cells showed lower levels of DNA methylation and histone H3 acetylation, development to blastocyst stage was decreased. The epigenetic markers of embryos cloned from 0.01 μM 5-aza-dc remained unchanged.

**Conclusion:**

These results show that 5-aza-dc is not a suitable choice for modifying nuclear reprogramming. Finally, it was concluded that the wide genomic hypomethylation induced by 5-aza-dc deleteriously impacts the developmental competency of cloned embryos.

## Introduction

Successful reprogramming of differentiated somatic cells to compel them back to the state of totipotency capable of embryonic development, via a process so called somatic cell nuclear transfer (SCNT), has been demonstrated by the birth of cloned offsprings in several species (1-10). However, the overall efficiency of SCNT as based on the percentage of viable cloned offspring or number of embryos transferred has ranged between 1-5%, far below that commonly reported for fertilized embryos (45-60%) (11-13). Although the exact reason(s) of this great discrepancy is not completely understood, several lines of molecular evidence indicate that aberrant epigenetic modification of donor cell during nuclear reprogramming in the SCNT procedure can be regarded as the main cause of reported failure. This occurs due to the abnormal reactivate expression of the embryonic genome (14-16). In this regard, analysis of the gene expression profile has revealed that over 30% of cloned mice embryos failed to express the complete gene set. In particular Oct-4, an essential pluripotency gene for the production of embryonic stem cells (ESCs) cells, failed to be re-expressed in a large number of somatic clones (17-19).

Among different characteristics that distinguish somatic cells, methylation of about 70% to 80% of the genome is the most important component that appears to be the most important milestone in the way of cloning efficiency. Indeed, a successful program of cloning greatly depends on the extent of which these highly methylated regions of donor cell genome can be cleaned off. Therefore, it seems that pre-NT lowering of methylation levels of a nuclei donor cell may increase the chance of appropriate reprogramming during the SCNT procedure and therefore may enhance development of cloned embryos. 5-aza-2΄-deoxycytidine (5-aza-dc) is a synthetic substance routinely used as an anti-tumor drug which acts via inhibition of DNA methyl transferase. 5-aza-dc has also been used for induced global hypomethylation of donor cells destined to SCNT and early stage cloned embryos (20- 24). However, the effect of 5-aza-dc treatment of donor cells on the pattern of pluripotency gene expression of the resultant cloned embryos has not been elucidated. Moreover, great variations have been observed between the percentages of cloned blastocysts developed in each study, which precludes precise comparison between them. Through a high output and reproducible zona-free method of SCNT initially described by Oback et al. (25), we investigated the effect of 5-aza-dc treatment of donor cells on *in vitro* developmental competence of cloned bovine embryos. In addition, the pattern of Oct-4 gene expression was evaluated by semi-quantitative reverse transcriptase-polymerase chain reaction (RT-PCR) analysis in individual SCNT blastocysts as compared to control SCNT and *in vitro* fertilization (IVF) embryos.

## Materials and Methods

All chemicals were obtained from Sigma Aldrich Chemicals (St. Louis, MO, USA) and all media were obtained from Gibco, Invitrogen Corporation (Grand Island, NY, USA), unless otherwise indicated. This study received the approval of the Ethics Committee of Royan Institute.

### Somatic cell preparation and 5-aza-dc treatment

A skin biopsy taken from the ear of a healthy adult bull was used for the *in vitro* explant culture of somatic cells according to the method described by Ashtiani et al. (26) At passage 1, a portion of cells was subjected to immunostaining against intermediate filaments vimentin and pancytokeratin to distinguish fibroblasts from epithelial cells, respectively (27). Confirmed fibroblast cells were propagated until passage 3 and then cells at a density of 1×10^5^ were added to 30 mm culture dishes containing 2 ml of culture medium [DMEM/F-12 plus 10% fetal calf serum (FCS)]. For 5-aza-dc treatment, cells in each culture dish were subjected to one of the concentrations of 5-aza-dc: 0.0 μM (control), 0.01 μM, 0.08 μM, 0.3 μM and incubated for 72 hours at 38.5 ºC, 5% CO2 and maximum humidity.

### Recipient oocyte preparation and zona removal

Abattoir derived cow ovaries were used as the source of immature cumulus oocyte complexes (COCs). The procedure of *in vitro* maturation (IVM) was as described elsewhere (28). At 20-22 hours post IVM, matured oocytes were cleaned off from surrounding granulosa cells by manual pipetting in 0.1% hyalorunidase dissolved in Hepes tissue culture medium 199 (HTCM) plus 10% FCS. Denuded oocytes were thereafter washed thoroughly in HTCM and then pools of 25-30 oocytes were incubated for up to 1 min in HTCM containing 10% FCS and 5mg/ml protease (p-8811). After 1 minute, oocytes with completely or partially dissolved zona pelucida (zona) were transferred back into HTCM and 20% FCS devoid of protease for up to 3 minutes, until the zona removed completely and the oocytes return back to their spherical shape. Rested oocytes were then thoroughly washed in HTCM plus 10% FCS for complete enzyme neutralization.

### Somatic cell nuclear transfer

**Fig 1:**
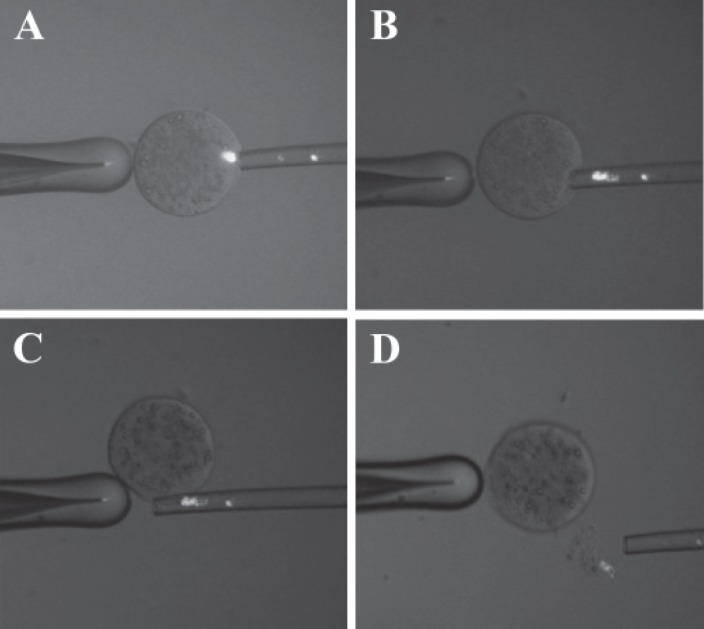
A. Matured oocyte (at MII stage) with maternal chromosome that stained with Hoechst under UV light with an enucleating pipette (perpendicular break) adjusted near to the MII Plate at 3 oclock, B. MII spindle is suckling into enucleating pipette, C. Separation of cytoplast and karyoplast by a brief kick on the warm stage, D. Completely removed karyoplast replacing a cytoplast

The entire procedure of oocyte emulation was performed in a basic medium comprised of phosphate buffer saline (PBS) free of Ca^2+^ and Mg^2+^, 20% FCS, Na pyruvate (2 mg/ml), bovine serum albumin (1 mg/ml) (BSA), poly vinyl alcohol(1 mg/ml) (PVA), and glucose (0.036 mg/ml). For enucleation, zona-free oocytes were incubated in basic medium containing 5 μg/ml Hoechst 33342 stain for 5 min before being transferred into separate microdroplets of basic medium on the pre-warmed microscopic stage. Under 100% magnification and constant ultraviolet exposure, each oocyte was moved using a blind separation pipette and a blunt perpendicular break enucleation pipette (15-20 μm inner diameter) until the metaphase plate of the MII oocyte was clearly observed at the 3 o΄clock position close to the enucleation pipette and at the same focal plane. The MII chromosome was then gently aspirated into the enucleation pipette and separated from the whole cytoplast by a brief kick by the fingers on the microscopic stage (Fig 1).

For nuclear transfer, somatic cells from treatment and control groups were trypsinised, washed by centrifugation (700 g) and then resuspended in HTCM plus 0.5% FCS. Aliquots of approximately 20 to 50 individual cells were then prepared in HTCM+0.5% FCS droplets that already contained 10 μg/ml phytoheamoglutinin (PHA). A group of 5 to 10 enucleated oocytes were then added into each droplet and then each oocyte was gently pushed over a single cell as the oocyte and cell adhered to each other. Oocyte-donor cell couplets were further incubated in PHA droplets for 5 to 10 minutes.

For electrofusion, groups of 10 to 15 couplets were washed and incubated for up to 3 minutes in fusion buffer (0.2 M mannitol, 100 μM MgSO_4_, 50 μM CaCl_2_, 500 μM Hepes, 0.05% BSA) and transferred into the fusion chamber (0.5 mm apart) that was filled with fresh fusion buffer. After a brief manual alignment, couplets were completely aligned by sinusoidal current (1000KHZ, 7 V/cm) and then fused with two direct currents (1.75 KV/ cm, 30 μ second each and 1 second delay within) as described elsewhere (29). Fusion was performed at room temperature (RT) and pulsed couplets were turned back into wash and rest drops (TCM plus 10% FCS). Thirty minutes post- fusion, fusion efficiency was assessed and non-fused and lysed couplets were discarded. Two to 4 hours post-fusion, NT units were further activated by a 5 minutes incubation in 5 μM calcium ionophore followed by a 4 hours incubation in 6-dimethyl aminopurine (DMAP) as described by Hosseini et al. (29). Activated zona-free reconstructs were then cultured in groups of seven in wells drained in 25 μl of serum- free continuous formulation of modified synthetic oviductal fluid (mSOF) for eight days when the percentages of reconstructed cleaved and further developed into blastocysts. Blastocysts were counted and used for various assessments: 1. differential staining to detect total cell number (TCN), inner cell mass (ICM) and ICM/TCN ratios as described elsewhere (30), 2. immunofluorescence staining to detect DNA methylation and histone acetylation, and 3. semi-qualitative RT-PCR to detect Oct-4 gene expression as described below.

### Blastocyst assessments

#### Differential staining

Total cell number (TCN), inner cell mass (ICM) and ICM/TCN ratios of the developed blastocysts were performed as described by Forouzanfar et al. (30).

#### Immunofluorescence staining

Immunofluorescence staining analysis was performed to detect the levels of methylated CpG islands on DNA and acetylated histone H3 on lysine 9 (H3K9) in developed blastocysts. For this purpose, blastocysts were thoroughly washed in PBS containing 1mg/ml PVA, fixed in 4.0% paraformaldehyde (PF) for 30 minutes followed by permeabilization in 0.5% triton X-100 for 15 minutes.

To stimulate reaction between DNA 5-methyl cytosine and corresponding antibody, embryos destined for the DNA methylation assay were further treated with 4N HCl for 60 minutes at RT as a treatment denaturing DNA into the singlestranded form. After washing with PBS-, embryos were treated with 3% BSA in PBS- for 60 minutes at RT to preclude un-specific binding of primary antibody.

These embryos were then incubated with primary antibody which was either mouse monoclonal anti-5-methyl cytosine for DNA methylation (Eurogentec, BI-MECY-0100) or mouse monoclonal anti-H3K9 for histone acetylation (Sigma H0913) for 1 hour at 37ºC. Blastocysts were then washed with PBS containing PVA and subsequently incubated with fluorescein isothiocyanate (FITC)- conjugated goat anti-mouse IgG secondary antibody (CHEMICON AP124F) for 60 minutes at 37°C. Embryos were also stained with propidium iodide as the counterstain. After mounted on microscopic slides and covered with coverslips, the intensities of acetylation and methylation were imaged [×400, epiflourescence microscope (Olympus, BX51)] and analyzed by Image J software (National Institute of Mental Health, Bethesda, Maryland, USA).

### Semi-quantitative RT-PCR

Total RNAs of individual cloned embryos were prepared using an RNeasy® Micro Kit (Qiagen, Cat.No.74004). cDNA was synthesized from 200 ng RNA which was incubated with a random hexamer primer with the RevertAidTM First Strand cDNA Synthesis Kit (Fermentas). For reverse- transcription, the reaction tubes were kept at 25°C for 10 minutes, 42°C for 60 minutes and 72°C for 10 minutes to inactivate the reaction. cDNAs were subjected to PCR using the specific primers for Oct-4 as a candidate for proper epigenetic reprogramming and pluripotency. Glyceraldehyde-3-phosphate dehydrogenase (GAPDH), as a housekeeping gene, was used as an internal control in gene expression analysis. The primers used were as follows: Oct-4, forward primer 5'-GGGACACCTCGCTTCTGAC- 3' and reverse primer 5' GGGGGCCGCAGCTTACAC- 3' 603bp; and GAPDH, forward primer 5'-GGCATCGTGGAGGGACTT- 3' and reverse primer 5'-GGAGGCCATGTCGACCA- 3' 496 bp. PCR reaction was performed using Ex Taq (cat. number Takara, Otsu, Japan). The RT-PCR conditions were as follows: denaturation at 94°C for 5 minutes, followed by 35 and 40 amplification cycles for GAPDH and Oct-4, respectively; denaturation at 94°Cfor 1 minutes; annealing at 55°C and 59°C for GAPDH and Oct-4, respectively for 1 minutes; and extension at 72°C for 1 minutes. The final cycle contained an additional extension at 72°C for 10 minutes. PCR products were visualized on 1.5% agarose gel under ultraviolet light. Image analysis of ethidium bromide-stained agarose gel was used to quantify gene expression levels. Semi- quantitative RT-PCR was performed using GeneTools from SynGene software (version 3.06, UK).

### Statistical analysis

The percentage data of cloned embryo development were modeled to the binomial model of parameters by ArcSin transformation. These transformed data, along with crude data of the cellular characteristics, were analyzed by one way ANOVA model of SPSS 17. Differences were compared by Tukey multiple comparison post hoc test. All data were presented as means ± SEM and differences considered as significant at p< 0.05.

## Results

### *In vitro* development of zona-free cloned embryos from 5-aza-dc treated donor cells

After treatment of donor cells with various concentrations of 5-aza-dc, we performed the zonafree nuclear transfer method using untreated and treated cells. As shown in table 1 there was no significant difference in cleavage rates between the treated and untreated groups. However the lowest cleavage rate belonged to the 0.3 μM 5-aza-dc treated cells. On the other hand, the progression of reconstructed embryos to the blastocyst stage decreased, which was insignificant for 0.01 μM (28.6 ± 5.3) and 0.08 μM (27.2 ± 11.2) 5-aza-dc, and significant for 0.3 μM (6.5 ± 1.5) 5-aza-dc, compared with the untreated group (32.1 ± 4.3) (p<0.05).

### Assessment of epigenetic status and quality of cloned embryos from 5-aza-dc treated donor cells

Levels of methylation on CpG islands on DNA decreased in cloned blastocysts derived from 5-aza-dc treated cells compared to untreated cells, and surprisingly the level of H3K9 acetylation in cloned blastocysts also decreased compared to the untreated cells (Table 2).

As shown in table 2 these changes on DNA methylation and histone acetylation were not significant for 0.01 μM 5-aza-dc yet significant for 0.08 and 0.3 μM 5-aza-dc compared to the untreated cells (p<0.05).

**Table 1:** *In vitro* development of cloned embryos generated by 5-aza-dc treated donor cells


			Embryo development	
		Activated oocytes (No.)	Cleavage (% ± SEM)	Blastocyst (% ± SEM)

**5-aza-dc (µM)**	**0**	175	156 (94.5 ± 2.1)^a^	50 (32.1 ± 4.3)^b^
	**0.01**	175	154 (97.5 ± 0.8)^a^	44 (28.6 ± 5.3)^b^
	**0.08**	104	92 (96.8 ± 2.9)^a^	25 (27.2 ± 11.2)^b^
	**0.3**	142	108 (87.1 ± 2.0)^a^	7 (6.5 ± 1.5)^a^


SEM: Standard error of mean Values with different superscripts within columns differ significantly at (p<0.05).

**Table 2:** Epigenetic status and quality of embryos generated by 5-aza-dc treated donor cells


		Embryo epigenesis			Embryo quality		
		No.	Acetylation ± SEM	Methylation ± SEM	No.	ICM	TCN

**IVF 5-aza-2-dc (μM)**		**10**	37.6 ± 2.1^b^	24.8 ± 2.9^b^	10	39.7 ± 2.8^c^	130 ± 5.6^c^
	**0**	**10**	49.6 ± 2.8^c^	35.6 ± 1.9^c^	15	31.1 ± 1.3^bc^	123.3 ± 7.6^c^
	**0.01**	**10**	46.9 ± 2.6^c^	32.7± 2.3^c^	10	37.7 ± 2.4^c^	125 ± 2.2^c^
	**0.08**	**10**	38.9 ± 2.6^b^	26.2 ± 2.5^b^	10	24.4 ± 4.0^ab^	103.3 ± 4.1^b^
	**0.3**	**5**	31.7 ± 2.9^a^	14.7± 2.7^a^	2	18.5 ± 2.8^a^	96.3 ± 5.1^a^


ICM: Inner cell mass TCN: Total cell number Values with different superscripts within column differ significantly (p<0.05).

As shown in Table 2 the mean of ICM and TCN cell numbers reduced which was significant in embryos cloned from 0.3 μM 5-aza-dc treated cells (p<0.05).

**Fig 2:**
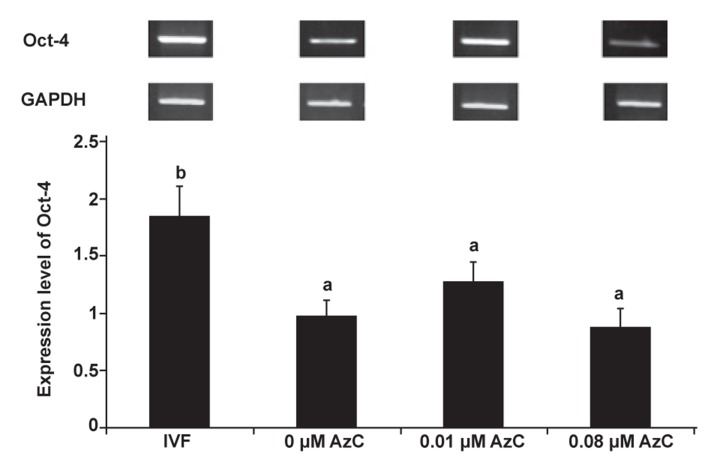
Semi-quantitative RT-PCR results for the analysis of Oct-4 gene expression in IVF and cloned blastocysts produced by 0, 0.01 and 0.08 μM 5-aza-dc treated cells (p<0.05).

### Effect of 5-aza-dc on gene expression on cloned embryos at the blastocyst stage

In order to assess the effect of different concentrations of 5-aza-dc on Oct-4 expression in cloned embryos, we evaluated the expression of GAPDH and Oct-4 by semi-quantitative RT-PCR at the blastocyst stage. Fig 2 reveals that expression of Oct-4 relative to GAPDH in cloned blastocysts derived from 0.01 and 0.08 μM 5-aza-dc treated cells increased, which was insignificant compared to cloned blastocysts derived from untreated cells whereas IVF embryos had significantly higher levels of Oct-4 expression in comparison with cloned embryos produced by various treated cells (p<0.05). Due to limited numbers and low quality blastocysts (Table 2) derived from 0.3 μM 5-aza-dc treated cells, RT-PCR was not performed for this group.

## Discussion

In SCNT, the transferred nucleus of somatic donor cells has specific epigenetic markers which are related to the type of tissue that the somatic cell is derived from. Somatic cells have high levels of DNA methylation which should be modified during nuclear reprogramming (16, 31). In relation to the high levels of DNA methylation in somatic donor cells, cloned embryos in the preimplantation stage show a global genomic hypermethylation that may be the major cause of the large number of developmental abnormalities observed in cloned embryos. This global DNA hypermethylation exhibits incomplete and insufficient nuclear reprogramming that is related to abnormal expression of key genes necessary for proper development.

In regard to this global genomic hypermethylation in NT embryos, modification of epigenetic markers in somatic donor cells before cloning with pharmaceutical agents may improve cloning efficiency. 5-aza-2΄-deoxycytidine (5-aza-dc), an anti-tumor drug that reduces levels of DNA methylation via inhibition of DNA methyl transferase enzymes, has been shown to cause overexpression of imprinting genes (32).

Previously, 5-aza-dc has been used to improve development of NT embryos. It has been shown that treating donor cells with high concentrations (>0.08 μM) of 5-aza-dc for 72 hours had deleterious effects on the developmental competency of NT embryos, while a lower concentration of 5-azadc (0.01 μM for 72 hours) insignificantly increased the blastocyst rate. (21-24).

In the present study, we investigated the effect of pretreated somatic donor cells with various concentrations of 5- aza-dc on development of NT embryos. We also assessed the effect of this treatment on expression of Oct-4, epigenetic markers (DNA methylation and acetylation levels of H3K9) and embryo quality.

As shown in table 1 treating donor cells with various concentrations of 5-aza-dc had no significant effect on cleavage rate. These results show that the cleavage rate has not been affected by alteration in epigenetic markers and may be a resultant of an optimized artificial activation procedure.

It has been shown that 5-aza-dc not only can reduce levels of DNA methylation but also has an indirect effect on histone acetylation and can increase it via recruitment of the histone acetyl transferase enzyme (HAT) (33). In this regard, we hypothesize that treating donor cells with 5-aza-2-dc makes epigenetic markers of donor cells resemble those which exist in natural fertilization, may improve cloning efficiency (14, 15, 34, 35). To induce hypomethylation in DNA, we treated donor cells for 72 hours (doubling time is approximately 24 hours). However, treating donor ells with 0.3 μM of 5-aza-dc had a harmful effect on the development of cloned embryos which significantly reduced the blastocyst rate, while in contrast to the aforementioned studies, 0.01 and 0.08 μM of 5-aza-dc insignificantly reduced the blastocyst rate (21, 22). This results show that possibly global demethylation disrupted gene expression of essential genes vital for embryo development. On the other hand, many researchers have reported the cytotoxic effects of 5-aza-dc on cells. Therefore, further experiments are required to test a DNA methyl transferase (Dnmt) inhibitor agent that is safer. Furthermore, the quality (ICM and TCN) of embryos reconstructed from 0.3 μM 5-aza-dc treated cells were significantly lower compared to other groups which was related to the cytotoxic effects of 5-aza-dc.

The epigenetic markers of reconstructed embryos from 0.3 μM 5-aza-dc, DNA methylation and histone H3 acetylation, significantly reduced compared to other groups. Despite a significant reduction in DNA methylation of these embryos, this treatment also significantly decreased blastocyst development; therefore, it seems this treatment is not a proper treatment for improving cloning efficiency.

The promoter of the Oct-4, an essential transcription factor for generating totipotency in an embryo, is rich in methylated CpG islands (36, 37) which inefficiently demethylate following nuclear transfer and therefore affect proper development in early embryos. This inefficient demethylation is a resultant of incomplete epigenetic reprogramming (38, 39).

The exclusion of Dnmt1o (Dnmt 1 oocyte) from the nucleus for one cell cycle caused passive methylation of the maternal genome. The endogenous Dnmt of the somatic donor cells is associated with the nucleus in all steps and causes genomic hypermethylation in clones. For this purpose we used 5-aza-dc treated cells to inhibit the action of Dnmt1s ( Dnmt 1somatic) to reduce the level of genomic methylation and make the donor genome more amenable for nuclear reprogramming. Following this treatment, semi-quantitative expression analysis showed that the level of Oct-4 in cloned blastocysts increased but was insignificant compared to the control group.

## Conclusion

After testing various concentrations of 5-azadc, we found that although treatment of the donor cell with 5-aza-dC alters epigenetic markers in cloned embryos similar to those found in IVF embryos, the developmental potential of these cloned embryos is also reduced. Finally these results suggest that perhaps wide genomic demethylation is harmful and not necessary for a proper nuclear reprogramming. Thus, maybe subtle changes in genomic methylation are more suitable for this purpose.
